# Dasatinib and FLAG-IDA Is an Effective Therapy for Initial Myeloid Blast Crisis but Involves a High Risk of Opportunistic Infections

**DOI:** 10.1155/2020/8867461

**Published:** 2020-11-11

**Authors:** Jorge Labrador, Gerardo J. Hermida, Rodolfo Alvarez, Victor Anso, Pilar de Vicente, Mercedes Goñi, Tomas Jose Gonzalez-Lopez

**Affiliations:** Department of Hematology, University Hospital of Burgos, Burgos, Spain

## Abstract

Blast crisis (BC) continues to be the major challenge in the treatment of chronic myeloid leukemia. Best results have been observed in a few patients who were successfully transplanted after returning to chronic phase. Recent studies focus on the combination of chemotherapy with imatinib, but results remain unsatisfactory. Since dasatinib induces deeper and faster responses, a reasonable strategy might be to combine it with chemotherapy, taking into account the alterations in T-cell response induced by dasatinib. However, there are no published studies or case reports supporting the use of dasatinib as first line treatment for initial myeloid BC, and very little is known about infectious complications associated with this drug. Based on this, we present the case of a patient diagnosed with an initial nonlymphoid phenotype BC, who achieved molecular response (MR^4.5^) with dasatinib and FLAG-IDA, but he suffered a pulmonary aspergillosis, CMV infection, and a CMV reactivation, prior to an allogeneic hematopoietic stem cell transplantation (HSCT). In conclusion, dasatinib and FLAG-IDA is an effective therapy for initial BC. However, a warning call is needed owing to the high risk of opportunistic infections, such as CMV.

## 1. Introduction

The outcome of patients diagnosed with chronic myeloid leukemia (CML) has improved considerably since the introduction of tyrosine kinase inhibitors (TKIs) in clinical practice. However, the prognosis of patients who progress to blast crisis (BC) remains extremely poor [1]. In addition, 10–15% of patients initially present with blast crisis.

According to blast lineage, BC is classified into lymphoid (20–30%) and myeloid (70%), with different treatment approaches [2]. While lymphoid BC often responds to treatment used for Ph + acute lymphoblastic leukemia, myeloid BC does not respond well to standard induction regimens used for acute myeloid leukemia [3].

Imatinib alone induces hematological responses in 24–32% of patients with myeloid blast crisis, including 2–8% of complete cytogenetic responses [4–7]. But, unfortunately, duration of these responses is very short [1]. The median response duration in different studies varies between 3 and 10 months (range, 1–43 months) [4–7].

Dasatinib induces 28% of hematological responses in these patients with a higher rate of complete cytogenetic responses (14–21%) [8]. Moreover, it has a faster response (between 1 and 2 months) and has the advantage that it crosses the blood-brain barrier. However, approximately half of patients relapse within 4 months (95% CI: 3.0–6.2 months), and median overall survival is only 8 months [8].

The best results have been observed in a few patients who could receive an allogeneic hematopoietic stem cell transplantation (HSCT) after returning to chronic phase [1]. Therefore, the objective must be to obtain a complete response or a second chronic phase to proceed with an allogeneic HSCT as soon as possible [1].

A reasonable strategy might be to combine TKIs with acute leukemia-like chemotherapy prior to HSCT to improve the prognosis of these patients. Some studies have shown better results when using various chemotherapy agents in combination with imatinib 600 mg daily [9], but these results remain unsatisfactory. Since dasatinib induces more profound and faster responses, it could be an effective option in combination with chemotherapy prior to allogeneic HSCT. In fact, dasatinib and FLAG-IDA has shown to be an effective and safety bridge to HSCT in a small study including 4 patients with BC transformation after failure with imatinib treatment [10]. However, there are no published studies or case reports that support the use of dasatinib as first line treatment for initial myeloid BC, that is, for patients who did not previously receive imatinib.

In addition, in spite of alterations in T-cell response induced by dasatinib, little is known about infectious complications in these patients.

Based on all these, a case is presented of a patient diagnosed with an initial nonlymphoid phenotype BC who achieved a molecular response (MR^4.5^) with dasatinib and FLAG-IDA but presented with high infectious toxicity including several opportunistic infections.

## 2. Case Presentation

**Figure 1:**
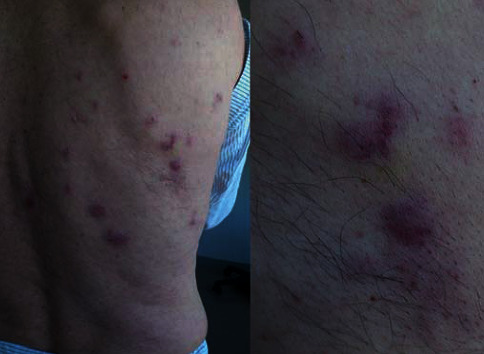
Erythematous, round, well-grouped plaques located at the rear right costal arch. A biopsy of the skin lesions was performed and reported as compatible with leukemia cutis.

**Figure 2:**
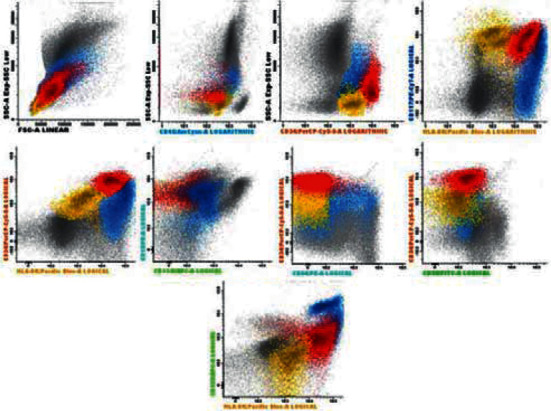
Flow cytometry at diagnosis showed 33% of blasts distributed in 3 populations. 1^st^ population, myeloid without differentiation (15%), in red: CD34++, CD117 +, HLA-DR +, CD45 + d, CD13+, CD64 −, CD35−/+ d, CD36−, CD33 +, CD71−/ +, CD105 + d, CD7−/ +, CD15−/ +, CD22−, CD38 +, CD123 +, CD4+d, and cMPO−. 2^nd^ population, dendritic (13%), in blue: CD34+d, CD117−/+, HLA-DR++, CD45+d, CD13 +, CD64−/ +, CD35−, CD36−/ +, CD33++, CD71−/+, CD105 + d, CD7−/+, CD15−/+, CD22−, CD38 +, CD123++, CD4 + d, and cMPO−/ +. 3^rd^ population, myeloid (5%), in yellow: CD34 + d, CD117 + d, HLA-DR−/+, CD45 + d, CD13 +, CD64−, CD35−, CD36−, CD33−, CD71−, CD105−, CD7−, CD15−, CD22 +, CD38−, CD123−, CD4−/+d, and cMPO−. Blasts were negative for CD10, CD16, CD11b, CD14, IREM-2, nTdT, CD56, CD19, 7.1, CD42, CD61, CD203c, cCD79a, cCD3, and CD3.

We present the case of a 67-year-old male patient, diagnosed with an initial nonlymphoid phenotype BC. At diagnosis, a physical examination revealed splenomegaly of 6 cm (splenomegaly was confirmed by abdominal ultrasound) and erythematous round well-defined grouped, not itchy, plaques located at the rear right costal arch (Figure 1). A biopsy of the skin lesions was performed and reported as compatible with leukemia cutis. Complete blood counts had values of hemoglobin 11.2 g/dL (13–17.5 g/dL), leukocytosis (WBC) 53.3 × 10^9^/L (4.5–11 × 10^9^/L) and platelets 48 × 10^9^/L (140–450 × 10^9^/L). A peripheral blood smear showed basophilia (3%), immature granulocytes (3% of bands forms, 2% of metamyelocytes, 10% of myelocytes), and 28% of blasts. Bone marrow aspirate showed granulocytic hyperplasia with 31% of myeloperoxidase negative blasts. Flow cytometry showed 33% blasts distributed in 3 populations (Figure 2): 1st, myeloid without differentiation (15%); 2nd, dendritic (13%); and 3rd, myeloid (5%).

Treatment was started with Hydrea until WBC <50 × 10^9^/L, and subsequently treatment began with fludarabine 30 mg/m^2^ i.v. days 1 to 4, cytarabine 2 g/m^2^ i.v. days 1 to 4, and idarubicin 10 mg/m^2^ i.v. days 1 to 3 (FLA-IDA). G-CSF was omitted due to hyperleukocytosis.

After that, the karyotype showed Philadelphia chromosome t (9; 22) (q34; q11) in all metaphases, and BCR-ABL fusion gene was evidenced by FISH and RT-PCR analysis with a break in the minor breakpoint region. Thereafter, the patient was diagnosed with myeloid-phenotype initial BC, and dasatinib was added at a dose of 100 mg initially and 140 mg thereafter.

On day +21, with recovered neutrophils but only 20 × 10^9^/L platelets, a bone marrow aspirate was performed to evaluate response, which showed 3% of blasts and negative minimal residual disease by flow cytometry (sensitivity 10–4). On day +41, due to hematological toxicity (grade 4 neutropenia and thrombocytopenia), a new bone marrow aspirate was performed which confirmed that the patient was still in complete response without platelets recovery, with negative minimal residual disease by flow cytometry, complete cytogenetic response, and major molecular response (detectable disease at a level of 0.024% by quantitative RT-PCR); moreover, both splenomegaly and cutaneous lesions disappeared. Therefore, dasatinib was temporally withdrawn for one week.

On day +47, neutrophil counts were recovered, but with platelets of 47 × 10^9^/L, a second dasatinib and FLAG-IDA cycle was started (including G-CSF 300 *µ*g/m^2^ s.c. on days 1–5).

**Figure 3:**
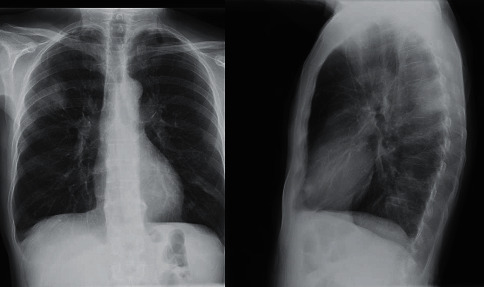
Chest X-ray showing a triangular consolidation in the right superior lobule.

During the second cycle, the patient developed bacteremia with *S. epidermidis* from his peripherally inserted central catheter (PICC). A few days after discharge, he was admitted to the hospital again due to probable aspergillosis in the right superior lobule. The chest X-ray was compatible with this (Figure 3) and with a positive galactomannan in the bronchoalveolar lavage, he was treated with oral voriconazole 200 mg/12 h. Of note, the absolute neutrophil count was normal.

On day +45, approximately 10 days after the bronchoalveolar lavage, the patient sought urgent medical attention, presenting with fever. He was asymptomatic, with hemodynamic stability, good performance status, and normal physical examination. ANC was 2.2 × 10^9^/L, so he was treated with empirical antibiotherapy, in accordance with the guidelines of our center, and continued with voriconazole. Both legionella and pneumococcal antigens were negative, as well as microbiological cultures. Since the patient remained febrile after 3 days of antibiotic treatment and had a questionable radiological worsening (on chest X-ray), it was decided to administer antifungal combination therapy consisting of voriconazole and liposomal amphotericin B at 5 mg/kg/d. On the seventh day, fever persisted despite empirical antibiotics and combined antifungal treatment, but the ophthalmogical investigation revealed no signs of fungal dissemination. In addition, galactomannan antigen was negative; however, CMV antigen was positive (5 infected leukocytes per 2 × 10^5^ leukocytes/mL). Therefore, it was concluded that the patient had a CMV infection, and the liposomal amphotericin B was discontinued. The CT scan was canceled and antiviral treatment was started with valganciclovir 900 mg every 12 hours, with good response.

It is noteworthy that, at 3 months of this CMV infection, the patient suffered a second CMV reactivation, which again responded to valganciclovir.

Besides infectious toxicity, the patient developed gastrointestinal toxicity (diarrhea), related to dasatinib, which required several admissions and temporary withdrawal of dasatinib with subsequent dose escalation to the maximum tolerated dose (100 mg/d).

The patient is currently in complete cytogenetic response, with molecular response (MR^4.5^), and was admitted for an allogeneic HSCT from a matched unrelated donor with a reduced-intensity conditioning.

## 3. Discussion

The efficacy of dasatinib and FLAG-IDA was demonstrated in our patient, who achieved a complete cytogenetic response after the first induction cycle and even subsequently improved (reaching MR^4.5^), which allowed the subsequent performance of an allogeneic HSCT. However, although the response was excellent, the significant infection toxicity associated with this regimen must be taken into account.

Apart from grade 3-4 neutropenia induced by dasatinib in 79% of patients with CML in advanced stages, requiring discontinuation of treatment in 60% of patients [11], which in our case was aggravated by the administration of chemotherapy, there are few data on infectious complications associated with dasatinib. Some *in vitro* studies suggest disturbance in the T-cell response [12–14], which has been confirmed in *ex vivo* studies [15]. Also, during treatment with dasatinib, there is a decrease in the number of B lymphocytes, CD4+ T lymphocytes, cytotoxic T lymphocytes, and natural killer cells [15] and inhibition of T-cell activation [15], and all these are dose-dependent [12]. These alterations in T cell immune response would involve an increased risk for reactivation of herpes family viruses (CMV, HHV-6, etc.) or other opportunistic infections. In this regard, there are several published clinical cases of viral reactivation following treatment with dasatinib [16]. Furthermore, it is noteworthy that in a small retrospective study of 16 patients who received dasatinib at a dose of 140 mg, 12 out of 16 patients experienced an infectious complication, including three opportunistic infections [15]. But it is not yet clear whether these viral reactivations observed in the literature, as in our patient, were due solely to the effect of dasatinib on T-cell response, or it was a cumulative effect on cellular immunity due to prior treatment with imatinib, or in our case, due to combination with fludarabine. However, it seems reasonable what some authors propose, suggesting prospective studies to evaluate the effectiveness of CMV monitoring in those patients treated with dasatinib [17], especially if they have additional risk factors as in our case, even outside the usual context of allogeneic HSCT.

In conclusion, dasatinib and FLAG-IDA is an effective therapy for initial BC. However, the high risk of opportunistic infections, such as CMV, should taken into account.

## Data Availability

All references included in this case report are indexed in PubMed clinical database.
